# Wave propagation in tunable lightweight tensegrity metastructure

**DOI:** 10.1038/s41598-018-29816-6

**Published:** 2018-07-31

**Authors:** Y. T. Wang, X. N. Liu, R. Zhu, G. K. Hu

**Affiliations:** Key Laboratory of Dynamics and Control of Flight Vehicle, Ministry of Education, School of Aerospace Engineering, Beijing Institute of Technology, Beijing, 100081 China

## Abstract

Lightweight metastructures are designed consisting of prismatic tensegrity building blocks which have excellent strength-to-weight ratio and also enable unique compression-torsion coupling. A theoretical model with a coupled axial-torsional stiffness is first developed to study the band structures of the proposed lightweight metastructures. Then, various unit cell designs are investigated for bandgap generations at desired frequency ranges. Broadband full-wave attenuation is found in the tensegrity metastructure with special opposite-chirality. Furthermore, tunable stiffness in the prismatic tensegrity structure is investigated and ‘small-on-large’ tunability is achieved in the metastructure by harnessing the geometrically nonlinear deformation through an external control torque. Prestress adjustment is also investigated for fine tuning of the band structure. Finally, frequency response tests on the finite metastructures are preformed to validate their wave attenuation ability as well as their wave propagation tunability. The proposed tensegrity metastructures could be very useful in various engineering applications where lightweight and tunable structures with broadband vibration suspension and wave attenuation ability are in high demand.

## Introduction

Tensegrity structures are lightweight spatial structures with a highly efficient material utilization and therefore, can form minimal mass systems with satisfying load-bearing capabilities^1–5^. Typically, tensegrity structures consist solely of bars and strings and own their shapes and stiffness to the prestress in the strings. As a result, the mechanical response of tensegrity can be easily adjusted by changing the topology of connections, masses’ shapes and positions as well as the prestress of the strings^6–9^. Such unique properties make tensegrities very desirable in various lightweight-emphasized structures in the fields of aerospace and civil engineering^10–13^. It’s noticed that such lightweight structures such as aerospace tensegrity structures are intrinsically affected by the low-frequency vibrations^9,11,14,15^. Even for small disturbances in the space environment, the tensegrity structure may suffer continuous vibration due to its small structural damping which makes the vibration control for the lightweight structures extremely challenging.

Elastic metamaterial is well known for its excellent low-frequency vibration suspension ability^16–18^. Inspired from metamaterial, metastructure has recently emerged to refer to a structure-like elastic metamaterial with excellent wave absorption abilities as well as stiffness-to-weight ratio^19,20^. Although tailoring the geometric and elastic properties of the metastructure’s building blocks could tune its wave behavior^21,22^, a broadband design still requires additional unit cells which inevitably increase the overall weight of the engineering structure^23^. One good solution for actively controlling the wave behavior of the metastructure is to introduce electromechanical coupling which provides an externally controllable degree of freedom in each unit cell^24–27^. Zhu *et al*. fabricated an adaptive metastructure with plastic tube and beam elements with surface-bonded piezoelectric patches and demonstrated that its bandgaps can be fully tailored by adjusting parameters of the shunted electric circuits. With the help of hardening and softening shunted circuits, tunable bandgap capacity as high as 45% was achieved experimentally^28^. However, it was also observed during the experiment that each shunted circuit requires independent adjustment due to the unavoidable inconsistency among manufactured metastructure’s unit cells, which could bring difficult in practical applications. The complicated stability condition in the control circuits could also become a problem to the robustness of the active metastructure^28^. An alternative solution to achieve tunable metastructure can be found without coupling with the other physical fields, which could significantly promote manufacturing feasibility of the unit cell as well as decrease the complexity of the entire system. Utilizing nonlinear elastic deformations, control of the small-amplitude linear wave in phononic crystals^29^ as well as LR-based elastic metamaterial^30^ have been demonstrated. Naturally, one can expect interesting and practically tunable elastic wave functions in tensegrity-based metastructure where geometric nonlinearity can be found intrinsically in those lightweight structures.

The nonlinear geometrical properties of tensegrity structures have been studied systematically^31–40^ and novel static/dynamic behaviors were discovered^41–48^. Oppenheim and Williams studied a tensegrity structure which demonstrated extreme stiffening-type response in the presence of rigid bases^41^. Amendola *et al*. developed new assembly methods for bi-material tensegrity and experimentally investigated its compressive response in the large displacement regime where switches from stiffening response to softening response were discovered^42^. Fraternali *et al*. studied the geometrically nonlinear behavior of uniformly compressed prismatic tensegrity structure (PTS) through full elastic and rigid-elastic models and both extreme stiffening and softening behaviors were observed^43^. It is noticed that the geometrical nonlinearity found intrinsically in the PTS is essential for the realization of these extreme mechanical responses. Moreover, the geometrical nonlinearity provides the basis for nonlinear wave propagation in a tensegrity array^44–46^. It is also noticed that by using the more practical full elastic model, the naturally coupled axial-torsional motions in a chiral-shape PTS can provide elastic responses that go beyond Cauchy continuum mechanics^47,48^ and therefore, create unique coupling wave modes with possible tunability through large-amplitude static loadings. Such tensegrity structures with rich coupling wave behaviors and potentially controllable dynamic properties are excellent building blocks to form metastructures for simultaneously lightweight and functional wave material systems.

In this paper, a full elastic model is developed to investigate the unique compression-torsion coupling in a PTS. Then, tunable stiffness and dispersion curves of a periodically-ranged PTS chain are observed under a torque-induced nonlinear deformation. Furthermore, various lightweight metastructure designs are investigated for bandgap generations at desired frequency ranges. Broadband isolation for simultaneous axial and torsional vibrations are observed in a metastructure with PTSs having opposite chirality. Moreover, tunable wave propagations are achieved in the proposed tensegrity metastructures by two approaches: (i) harnessing the geometrically nonlinear deformation of the PTSs under global control torque; (ii) adjusting the prestress in the strings for small-range and fine adjustment of the band structure. Finally, frequency responses of the finite metastructures under different loadings are numerically investigated to validate the band structure results.

## Results

### Theoretical model of tunable prismatic tensegrity structure

**Figure 1:**
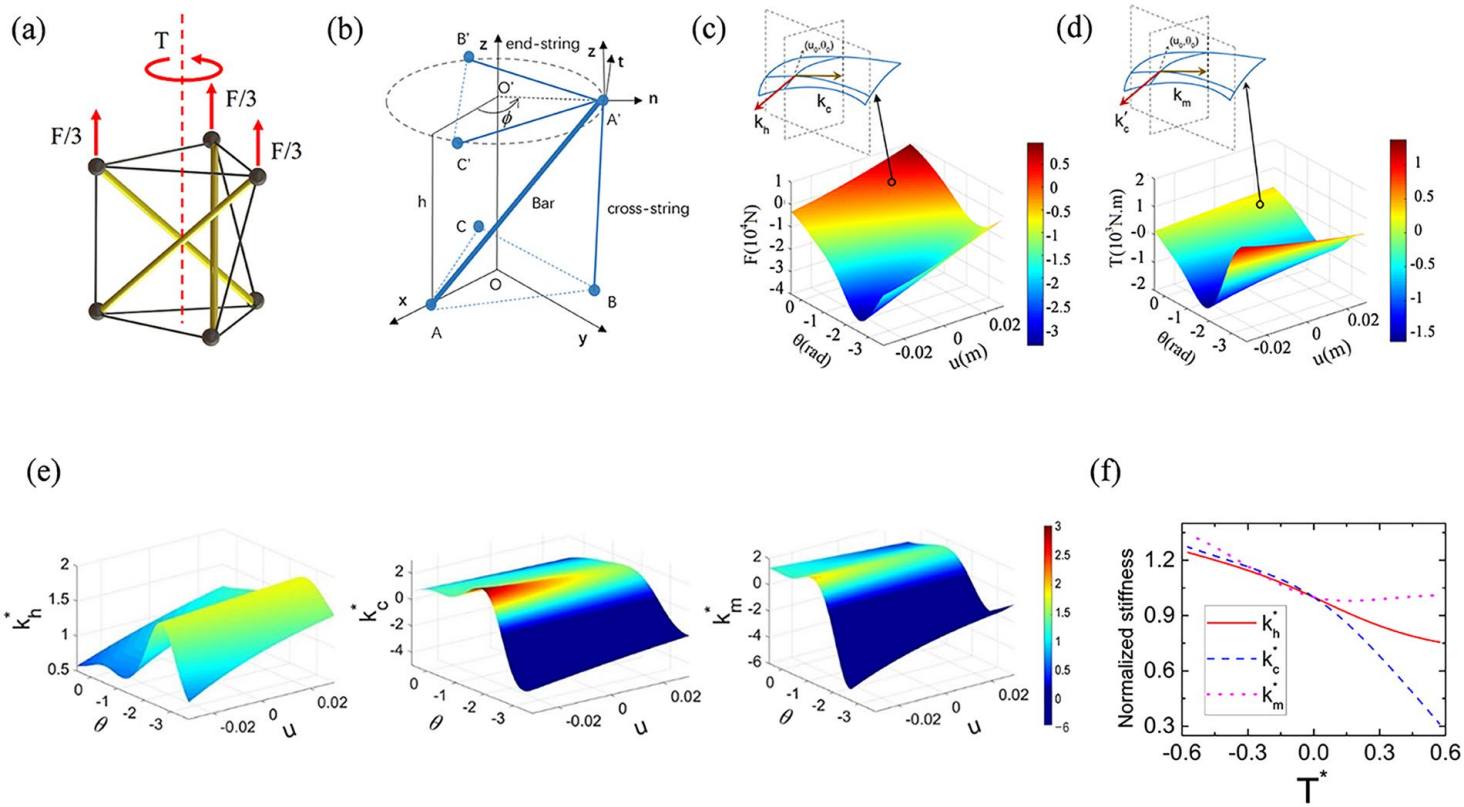
Configuration and geometric nonlinearity of the PTS. (**a**) Schematic of the PTS. (**b**) Reference configuration of the PTS. (**c**) *F* as nonlinear functions of *u* and *θ*. (**d**) *T* as nonlinear functions of *u* and *θ*. (**e**) ${k}_{h}^{\ast }$, ${k}_{c}^{\ast }$ and ${k}_{m}^{\ast }$ as nonlinear functions of *u*_0_ and *θ*_0_. (**f**) ${k}_{h}^{\ast }$, ${k}_{c}^{\ast }$ and ${k}_{m}^{\ast }$ change with the varying static torque T^*^.

Figure 1a shows the schematic of the PTS. It consists two parallel equilateral triangles at the top and bottom ends, which are then connected with three Nylon cross-strings (gray colored) and three polylactic acid (PLA) bars (yellow colored) in a right-handed chiral fashion. Both end-triangles are made of three inextensible end-strings and the gray spheres represent the spherical joints which permit rotational degrees of freedom (DOFs) of the bars and strings. A reference configuration of the PTS is also provided in Fig. 1b, where the displacement between the midpoint and the nodes of the equilateral triangles, the height of the PTS and the relative angle of the two equilateral triangles are *R*, *h* and *ϕ*, respectively. Under the axial or/and torsional loadings in Fig. 1a, both end-triangles maintain parallel with each other and the central axis of the PTS, OO′, is always along the *z* direction^41^. Unlike the previous studies which assume the rigidity of the bars^38,41,44^ and therefore, result in one-DOF systems, the elasticity of the bars (with PLA’s elastic modulus) are considered here for accurate PTS modelling as well as capturing the unique compression-torsion coupling effect. As a result, two DOFs, the relative rotational angle *θ* and the relative axial displacement *u*, between the two end-triangles are permitted in the studied tensegrity structure. By calculating the stable equilibrium condition of the PTS under zero external loadings, it can be found that only* ϕ* = 5π/6 is permitted. It is also noticed that the value of *θ* should be constrained within −7π/6 and π/6 to prevent any contact between the strings and the bars^41^. After calculating the potential energy of the system, the axial loading *F* and torque loading *T* as nonlinear functions of *u* and *θ* are plotted in Fig. 1c and d, respectively. The detailed derivations can be found in Methods section.

To demonstrate the tunability of the PTS, the effective tangent stiffness is first calculated by taking the partial derivatives of *T* and *F*, as shown in Fig. 1c and d. Figure 1e shows that the effective stiffness as nonlinear functions of *u*_0_ and *θ*_0_, where the subscript indicates a specific static loading condition. In the figure, the normalized stiffness is defined as ${k}_{h}^{\ast }$ = *k*_h_/*k*_h0_, ${k}_{c}^{\ast }$ = *k*_c_/*k*_c0_ and ${k}_{m}^{\ast }$ = *k*_m_/*k*_m0_ with *k*_h0_, *k*_c0_ and *k*_m0_ being the effective stiffness at the zero static loading condition (*u*_0_ = 0, *θ*_0_ = 0). In order to keep the tensegrity structure stable, ${k}_{h}^{\ast }$ and ${k}_{m}^{\ast }$ should always be greater than zero^36^ and therefore, the deep blue regions representing ${k}_{m}^{\ast }$ < 0 will not be studied in this research. Thanks to the intrinsic geometrical nonlinearity in the PTS, adjustable tangent stiffness can then be realized by applying different static loadings^49^, which consequently suggests an attractive approach for potential *in-situ* tuning of a wave system constructed by the PTS cells. Moreover, it is very interesting to notice that the effective stiffness changes more dramatically with the rotational angle than its changing with the axial displacement, which indicates that a static torque loading can be applied to the PTS as a more efficient way to adjust the PTS’s effective elastic properties and furthermore, control the dynamic behavior. Figure 1f shows the static torque adjustments on the ${k}_{h}^{\ast }$, ${k}_{c}^{\ast }$ and ${k}_{m}^{\ast }$, where normalization on the torque is applied as T^*^ = *T*/*k*_m0_. In the figure, ${k}_{h}^{\ast }$ and ${k}_{c}^{\ast }$ decrease monotonically when T^*^ increases while ${k}_{m}^{\ast }$ decreases when T^*^ < 0 but slightly increases when T^*^ > 0, which suggests different control strategies for the axial and torsional wave propagations in the PTS-based wave systems.

### Linear elastic wave propagation in a 1D infinite PTS chain

**Figure 2:**
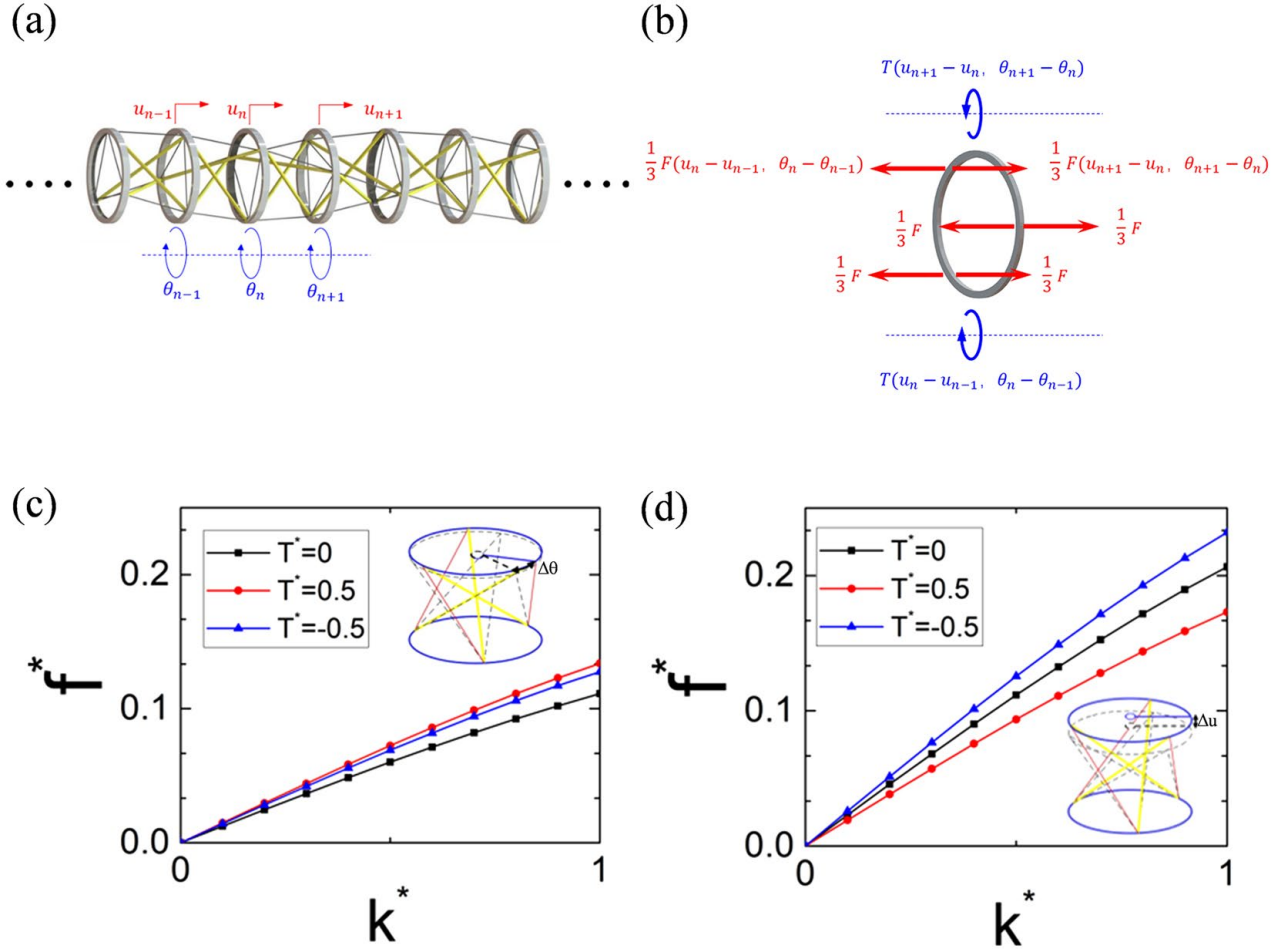
Schematic and dispersion curves of a 1D infinite PTS chain. (**a**) Schematic of a 1D infinite chain consisting of repetitive PTSs and hollow Ti disks. (**b**) Force analysis of the *n*^th^ hollow disk. (**c**) The dispersion curves of torsional wave propagation under different static torque loadings. (**d**) The dispersion curves of axial wave propagation under different static torque loadings.

A 1D infinite dynamic system is constructed by periodically arranging PTSs with same geometrical and elastic properties and connecting them with titanium (Ti) hollow disks which provide mass and moment of inertia for the dynamic system, as shown in Fig. 2a. Comparing with the mass of the metal hollow disk, the masses of the Nylon strings and PLA bars can be ignored. Moreover, due to the very high modulus of the hollow disk comparing with those of the string/bar, these disks are considered rigid in this study. Although nonlinear wave behavior can be found in the tensegrity system^43,44^, we here study the small-amplitude linear elastic wave propagation in the proposed 1D chain. Similar to the linear wave propagations in the soft and highly deformable phononic crystals^29,30^, modifications in the structural configuration of the tensegrity system can be realized by applying large external static loadings^50^, which vary the PTS’s effective stiffness at the corresponding equilibrium state and therefore, change small-amplitude linear elastic wave propagations.

By conducting equilibrium analysis in the unit cell (Fig. 2b) and using the Bloch theorem, the dispersion curves of the PTS system can be calculated by solving an eigenvalue problem and the details are described in Methods section. By applying different static torques to the PTS, various dispersion curves for torsional wave (rotational motion-dominated) and axial wave (axial motion-dominated) can be found in Fig. 2c and d, respectively. In the figures, the normalized frequency and normalized wavenumber are defined as f ^*^ = 2π*fh*_0_/(*k*_h0_/*k*)^1/2^ and k^*^ = *2kh/*π, respectively. Black lines are the dispersion curves when the chain is under zero external torque while the red lines and blue lines represent the dispersion curves when the chain is under positive (count-clockwise) and negative (clockwise) static torques. The inserted sketches in Fig. 2c and d demonstrate the mode shapes of the torsional and axial waves, respectively. For the torsional wave, both positive and negative static torque loadings increase the wave velocity due to the stiffening response of the PTS cell (pink dot curve in Fig. 1f). However, for the axial wave, positive static torque loading reduces the wave velocity due to the softening response of the PTS cell while negative torque loading increases the wave velocity due to the stiffening response of the PTS cell (red solid curve in Fig. 1f).

### Tunable bandgaps in the lightweight tensegrity metastructures

**Figure 3:**
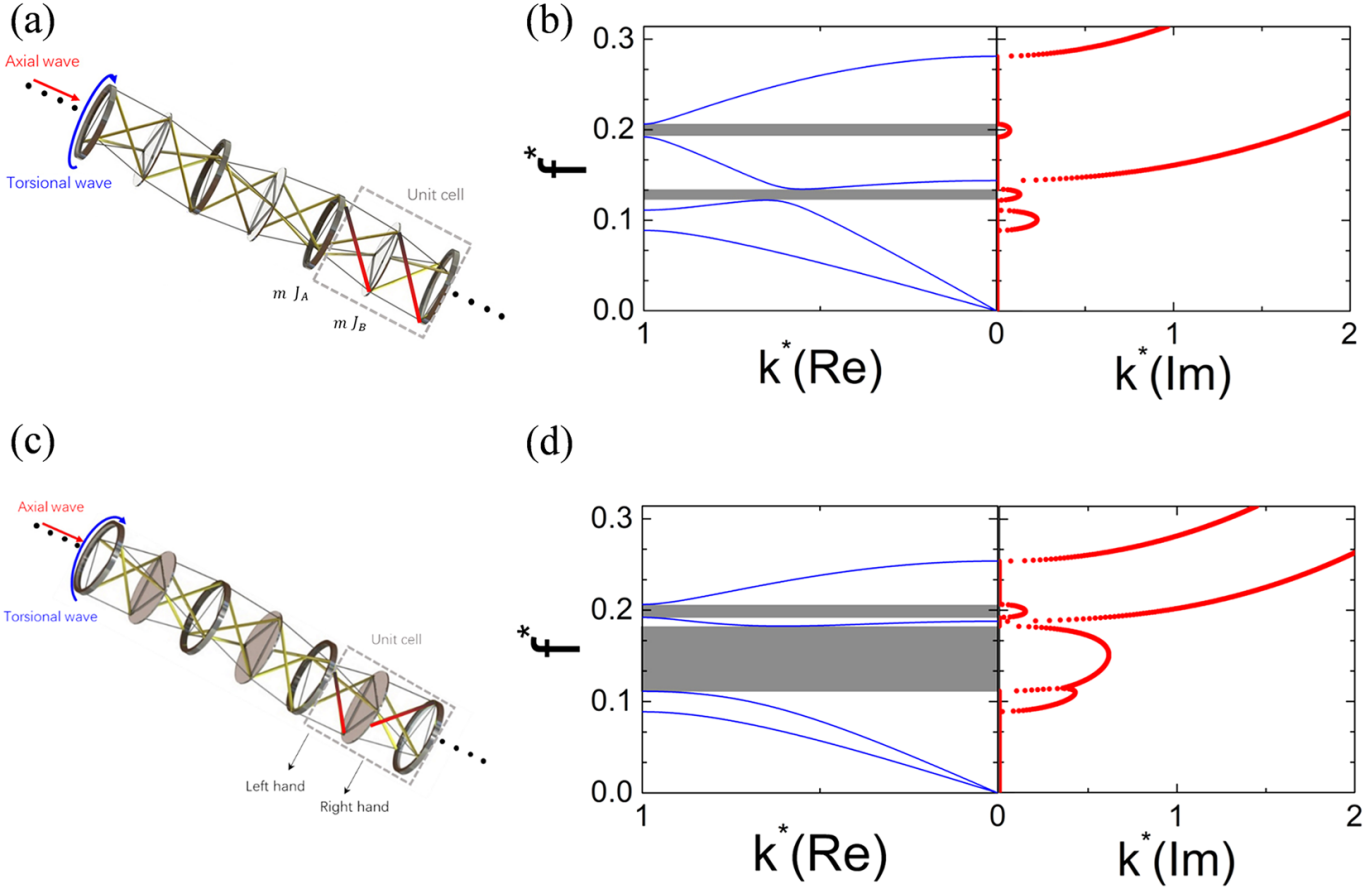
Schematics and dispersion curves of the tensegrity metastructures. (**a**) Schematic of a tensegrity metastructure with same chirality. (**b**) Dispersion curves of the tensegrity metastructure with same chirality. (**c**) Schematic of a tensegrity metastructure with opposite chirality. (**d**) Dispersion curves of the tensegrity metastructure with opposite chirality. Gray-shaded regions indicate full-wave bandgaps.

Various lightweight tensegrity metastructures are then designed based on Bragg scattering mechanism to create desired bandgaps at targeted frequency ranges. Figure 3a shows a tensegrity metastructure consisting of PTSs with alternating thick-hollow and thin-solid Ti disks. A unit cell of the tensegrity metastructure is highlighted inside the gray dashed rectangular region, which consists a solid disk (*m*, *J*_A_), a hollow disk (*m*, *J*_B_) and two sets of bars and strings that connect the neighboring disks with the same chirality (marked in red color). The moments of inertia of the hollow and solid disks are *J*_A_ = *J* and *J*_B_ = 1.81 *J* with the internal radius of the hollow disk being 0.9 *R*. The dispersion curves of the constructed tensegrity metastructure can be calculated as shown in Fig. 3b, where both real and imaginary parts of the normalized wavenumber are calculated for each frequency point. First, two full-wave bandgaps (neither torsional wave nor axial wave can propagate) are found in the gray-shaded regions with zero real wavenumbers and non-zero imaginary wavenumbers. The central-maximum curves of k^*^(Im) in both bandgaps indicate their Bragg-scattering origin^51–53^. Second, another central-maximum curve of k^*^(Im) is found in the frequency range just below the first full-wave bandgap where only propagating axial wave exists, which indicates a torsional wave bandgap generated by Bragg scattering. Third, two monotonically increasing curves of k^*^(Im) can be found above f ^*^ = 0.281 and f ^*^ = 0.144 which are the two cutoff frequencies for the axial and torsional waves, respectively.

Figure 3c shows the schematic of a tensegrity metastructure with opposite chirality in each unit cell. With oppositely chiral arrangements of the bars and strings (marked in red color) in neighboring PTSs, different effective stiffness is formed for the metastructure unit cell and Fig. 3d shows the calculated dispersion curves. First, it can be found that the first full-wave bandgap is greatly expended. The unchanged central-maximum profile of the k^*^(Im) curve still suggests the Bragg-scattering mechanism behind the enlarged bandgap. Second, the much larger k^*^(Im) values in the bandgap indicate stronger attenuations for both axial and torsional waves. Since neither the weight nor the size of the metastructure increases, the introduced opposite chirality proofs to be a practical way to efficiently attenuate elastic wave propagations in a much broader frequency range.

**Figure 4:**
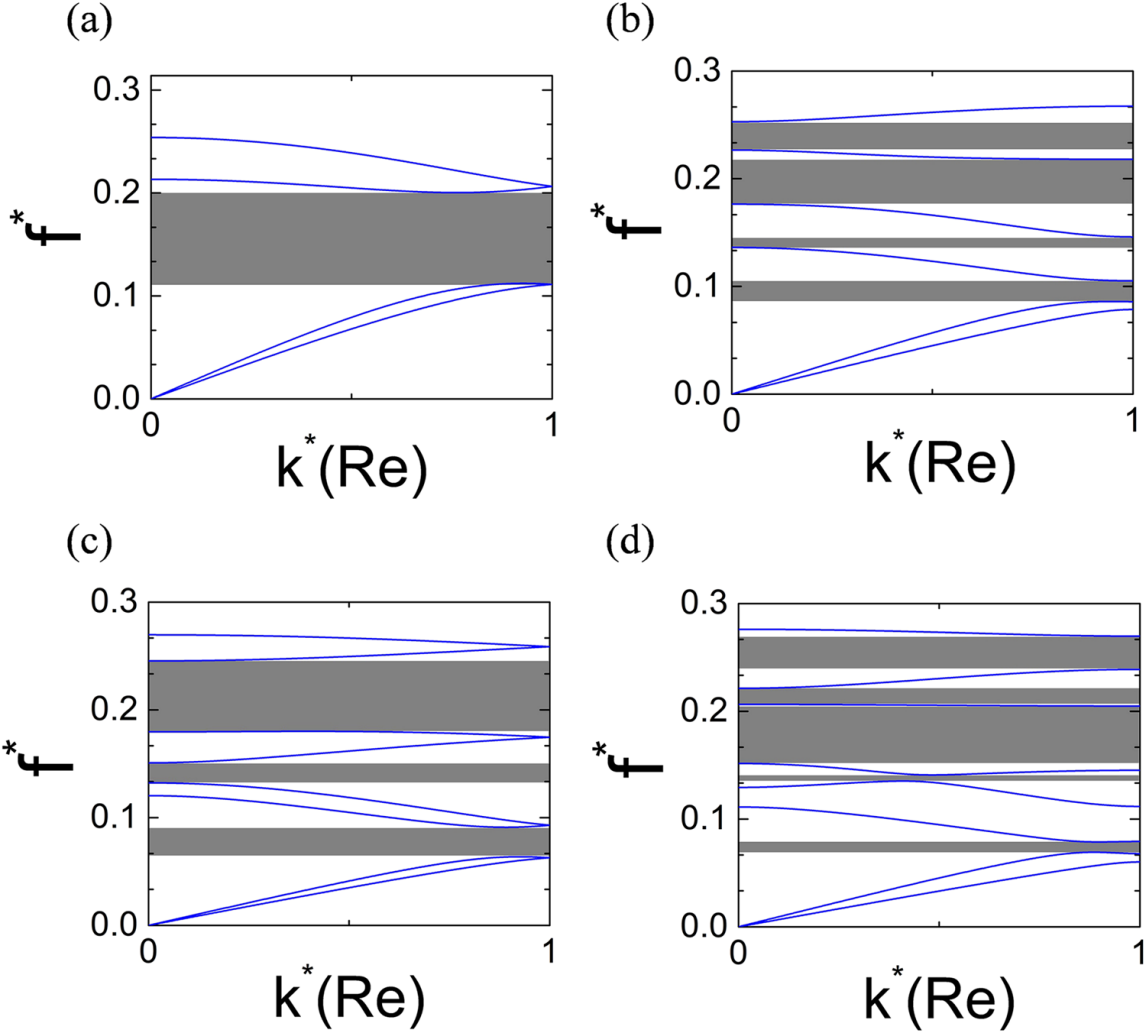
Dispersion curves of tensegrity metastructures consisting of hollow disks with different chirality combinations. (**a**) Tensegrity metastructure with + − super cell. (**b**) Tensegrity metastructure with − + − (or + − +) super cell. (**c**) Tensegrity metastructure with − − + + super cell. (**d**) Tensegrity metastructure with − − − + (or + + + −) super cell.

**Figure 5:**
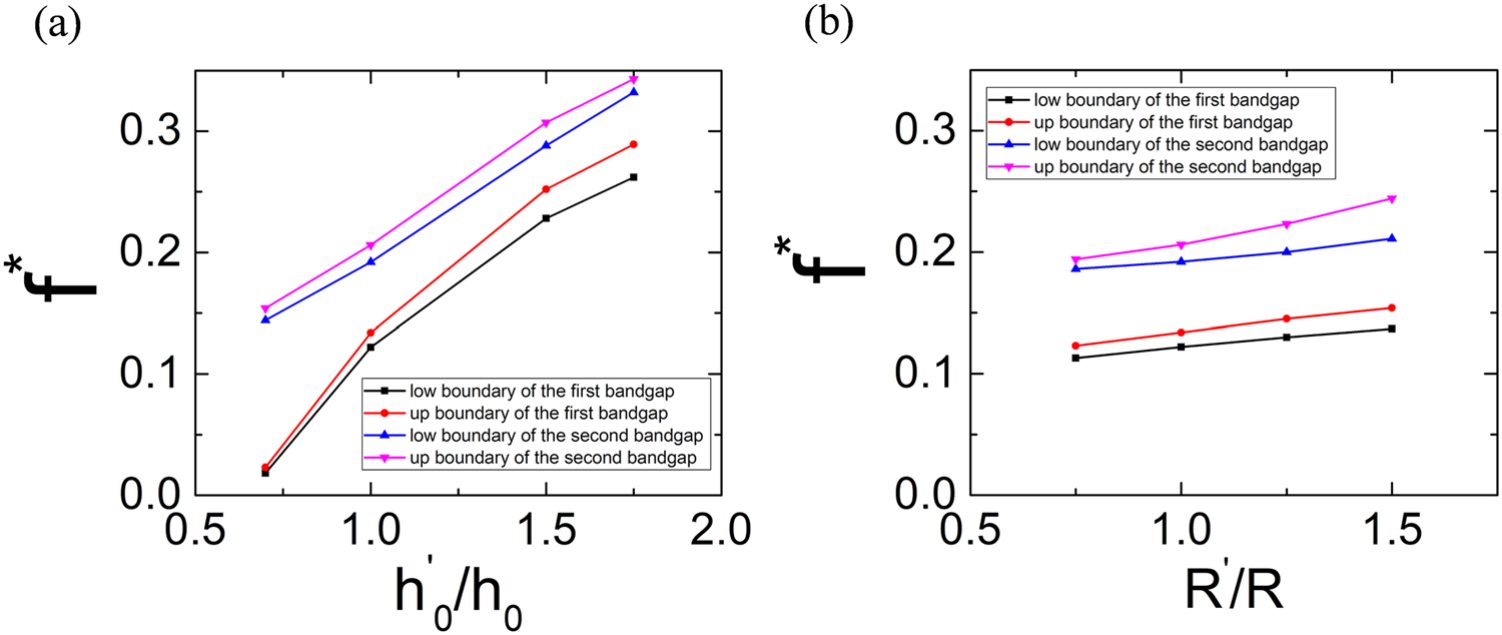
Relationship between the geometrical parameters of PTS and the bandgap frequencies. (**a**) Relationship between the initial height and the bandgap frequencies. (**b**) Relationship between the disk radius and the bandgap frequencies.

It is also interesting to investigate the dispersion curves of the tensegrity metastructures with only hollow disks but different chirality combinations. Figure 4 shows the calculated results with all possible chirality combinations in 2-cell, 3-cell and 4-cell super cells. The left hand and right hand chirality are signified as the symbol + and −, respectively. Figure 4a shows the dispersion curves of the metastructure with 2-cell (+ − type) super cell and single full-wave bandgap can be found in the gray-shaded region. For the super cells with 3 or 4 cells, multiple bandgaps can be found in Fig. 4b–d. It’s also noticed that by careful design of the super cell (− − + + type), the lowest boundary of the bandgap can be 33% lower than that of the 2-cell super cell case in Fig. 4a, as shown in the Fig. 4c. In Fig. 4d, the total width of the multiple bandgaps obtained from − − − + or + + + − type supercell reaches 137% of that of the single bandgap in Fig. 4a. Furthermore, the investigations on the bandgap behaviors with different initial height and different disk radius of the PTS are performed and the results are shown in Fig. 5. In these investigations, the masses of hollow and solid disks keep unchanged. In Fig. 5a, it can be found that the dimensionless frequencies of bandgaps increase monotonously with the dimensionless initial height ${h}_{0}^{^{\prime} }/{h}_{0}$. Figure 5b shows the relationship between the displacement between the midpoint and the nodes of the equilateral triangles of PTS, $R^{\prime}$, and frequencies of bandgaps. It can be found that the dimensionless frequencies of bandgaps and the width of the second bandgap increase monotonously with the dimensionless parameter $R^{\prime} /R$.

**Figure 6:**
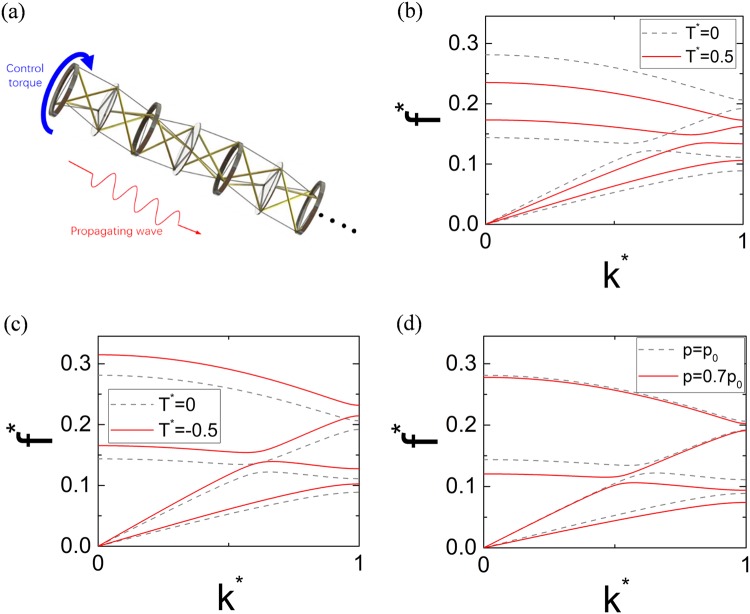
Schematic of small-on-large tunability in the tensegrity metastructure and the tunable dispersion curves achieved by the two approaches. (**a**) Schematic of small-on-large tunability in the tensegrity metastructure. (**b**) Dispersion curves of the tensegrity metastructure under positive control torque. (**c**) Dispersion curves of the tensegrity metastructure under negative control torque. (**d**) Dispersion curves with different prestresses in the strings.

As the tunability on single PTS’s effective tangent stiffness and elastic wave propagations in infinite PTS chain has been demonstrated previously, the following question arises naturally: Can the band structure of the tensegrity metastructure be controlled by an external loading? The key to answer this question lies on the recently demonstrated ‘small-on-large’ tunability^29,30^. The ‘large’ geometrically nonlinear deformation induced by a static control torque changes the effective stiffness of each PTS, the fundamental building block of the tensegrity metastructure, and therefore, affects the ‘small’ amplitude linear elastic wave propagation inside the metastructure, as shown in Fig. 6a. Both positive and negative control torques can be applied to the metastructure and their effects on the dispersion curves are shown in Fig. 6b and c, respectively. By imposing a positive control torque, the lowest bandgap rises to a higher frequency region while the cutoff frequency of the system drops, as shown in Fig. 6b. More interestingly, the second bandgap closes at around *f* ^*^ = 0.2 showing that the control torque can also be used to turn on/off the bandgap of the tensegrity metastructure, which will be further validated in the finite metastructure analysis. By imposing a negative control torque, not only the lowest bandgap but also the second bandgap as well as the cutoff frequency rise to higher frequency regions.

The adjustment on the prestress in each string of the PTS also provides an alternative way to tune the dispersion curves of the tensegrity metastructure. Prestress control in tensegrity structures has already been demonstrated by using a hydraulic actuator^54^. Figure 6d shows the dispersion curves of a tensegrity metastructure with different prestresses in the strings. It can be found that decreasing the prestress barely changes the dispersion curves at the high frequency region. But it can compress dispersion curves below *f* ^*^ = 0.15 and move the first full-wave bandgap slightly to a lower frequency region, which suggests a fine tuning approach to the dynamic system.

### Vibration isolation in the finite tensegrity metastructures

**Figure 7:**
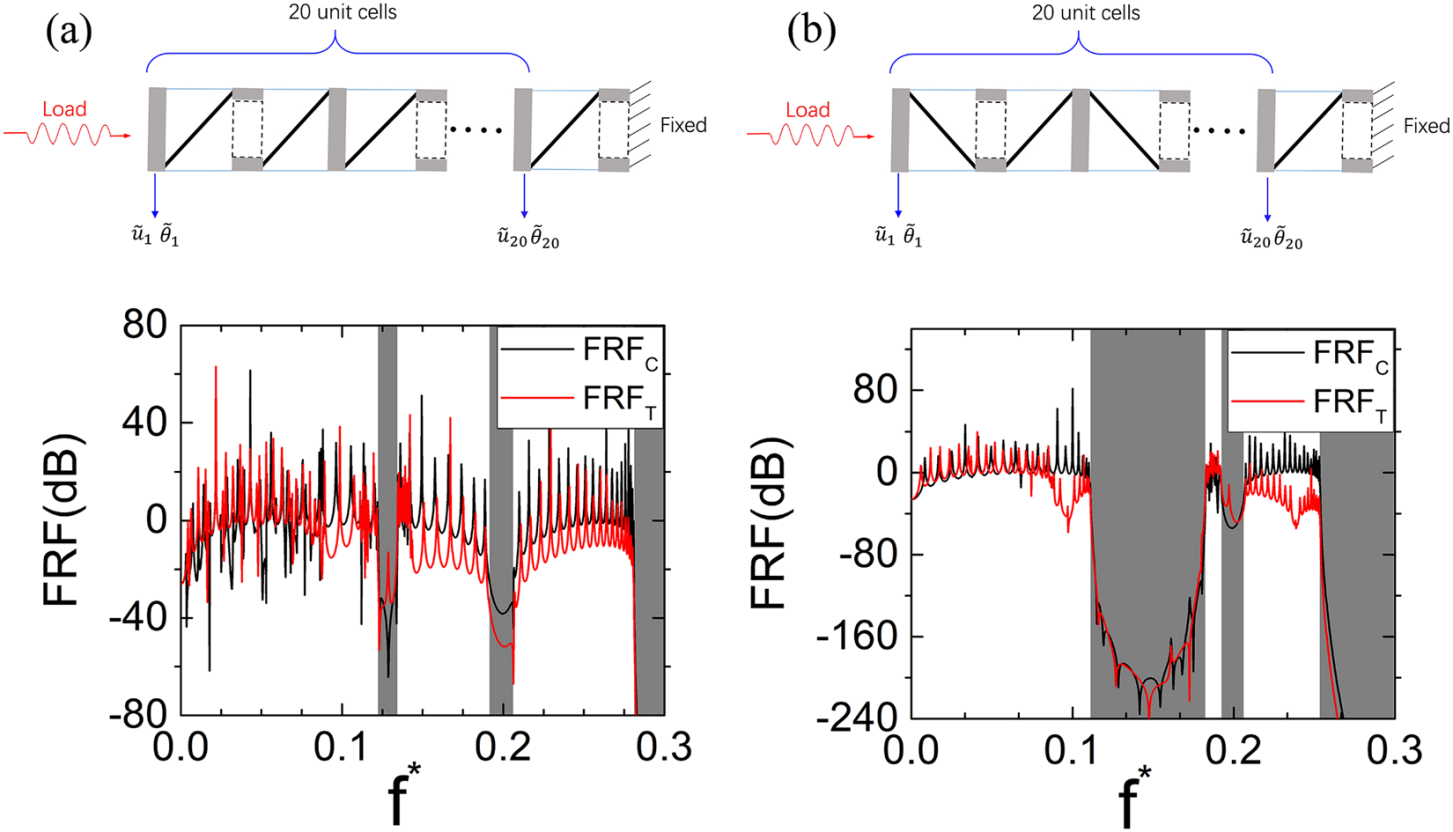
Schematics and FRF results of the finite tensegrity metastructures. (**a**) Schematic and FRF results of a finite tensegrity metastructure with 20 same-chirality unit cells. (**b**) Schematic and FRF results of a finite tensegrity metastructure with 20 opposite-chirality unit cells.

Previously, linear elastic wave propagations have been systematically investigated in the infinite tensegrity metastructures. However, infinite structures are not realistic in the real-world engineering applications and therefore, vibration tests in the finite structures with defined boundary conditions should be conducted to characterize as well as validate the dynamic properties of the proposed tensegrity metastructures. The top parts in Fig. 7a,b show the schematics of the finite tensegrity metastructures with same-chirality unit cells, opposite-chirality unit cells, respectively. Only side views are shown in the schematics with the blue thin lines, black thick lines being the strings connecting PTSs, the elastic bars, respectively. The solid, hollow disks are presented by the gray solid rectangles, the dash-line hollow rectangles, respectively. 20 unit cells are used in all three tensegrity metastructures. Harmonic axial and torsional force excitations are applied to one side of the metastructure while the other side is fixed. A frequency sweep is conducted in the normalized frequency range f ^*^ = (0, 0.34). Frequency-response functions (FRFs) are defined for the axial and torsional waves as FRF_C_ and FRF_T_, respectively. For steady-state vibrations, the FRFs for the two wave modes can be defined as ${{\rm{FRF}}}_{{\rm{C}}}=20\,\mathrm{log}({u}_{20}/{u}_{1})$ and ${{\rm{FRF}}}_{{\rm{T}}}=20\,\mathrm{log}({\theta }_{20}/{\theta }_{1})$, respectively, with ${u}_{j}={\tilde{u}}_{j}{e}^{-i\omega t}$ and ${\theta }_{j}={\tilde{\theta }}_{j}{e}^{-i\omega t}$ (*j* = 1 or 20) being the axial and torsional displacements measured at the sensor points located at first end disks of the 1^st^ and 20^th^ metastructure unit cells.

The bottom parts in Fig. 7a,b show the FRF_C_ and FRF_T_ results for the two finite tensegrity metastructures. The gray-shaded zones represent the full-wave bandgaps obtained from the infinite metastructure analysis, respectively. A vibration attenuation threshold is defined at −20 dB for both FRF_C_ and FRF_T_ to identify the frequency range of the attenuation zone in the finite metastructure^23^. In Fig. 7a, two normalized frequency ranges f ^*^ = (0.122, 0.134) and (0.192, 0.206) are found to have both FRF_C_ and FRF_T_ below −20 dB, which almost coincide with the two gray-shaded zones and therefore, validate the predicted full-wave bandgaps in the infinite metastructures. Apparently low amplitude of the FRF_T_ curve can be found in a small frequency range just below the first gray-shaded zone as well as the frequency range above f ^*^ = 0.144, which is due to the Bragg-scattering-induced torsional wave bandgap and the cutoff frequency of the torsional wave at f ^*^ = 0.144. It should be noticed that the axial waves with small coupled torsional motions are not affected in these two frequency ranges and still contribute to the FRF_T_ whose amplitude is therefore, above −20 dB. After the cutoff frequency at f ^*^ = 0.281, neither FRF_T_ nor FRF_C_ is above −20 dB. In Fig. 7b, the normalized frequency ranges f ^*^ = (0.111, 0.182) and (0.192, 0.206) are found to have both FRF_C_ and FRF_T_ below −20 dB, which also coincide well with the two gray-shaded full-wave bandgaps in the infinite opposite-chirality tensegrity metastructure. Both FRF_T_ and FRF_C_ are close to −240 dB at the central frequency point in the first attenuation zone, which can be well explained by the much larger k^*^(Im) value at the same frequency point in Fig. 3d. Also, low-amplitude FRF_T_ curves can be found in the torsional wave bandgap and above the torsional wave cutoff frequency at f ^*^ = 0.21.

**Figure 8:**
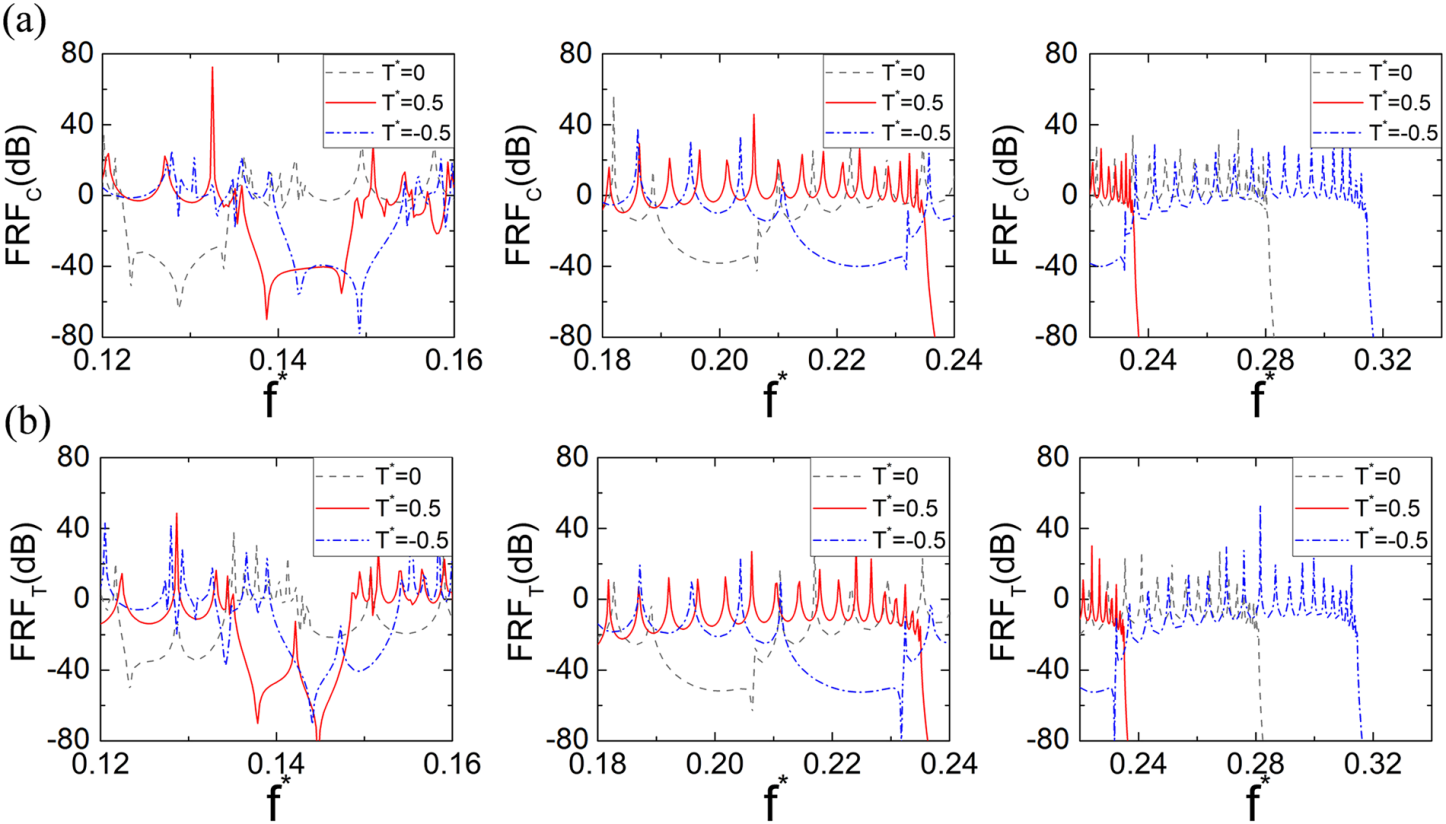
FRF results of the finite tensegrity metastructure with 20 same-chirality unit cells under different control torque loadings. (**a**) FRF_C_ results of the finite tensegrity metastructure with 20 same-chirality unit cells under different control torque loadings. (**b**) FRF_T_ results of the finite tensegrity metastructure with 20 same-chirality unit cells under different control torque loadings.

In order to validate the ‘small-on-large’ tunability in the proposed tensegrity metastructure, Fig. 8 shows the FRF results of finite tensegrity metastructures with same-chirality unit cells under different external control torques. In the figure, the gray dash lines, the red solid lines and the blue dash-dot lines are the FRFs with zero torque (T^*^ = 0), positive torque (T^*^ = 0.5) and negative torque (T^*^ = −0.5), respectively. Figure 8a and b demonstrate the result of the axial wave (FRF_C_) and torsional wave (FRF_T_), respectively. First, it can be found that the first valleys of the FRF_C_ and FRF_T_ curves (FRFs ≤ 20 dB) move from the frequency range f ^*^ = (0.111, 0.182) at T^*^ = 0 to higher frequency ranges f ^*^ = (0.135, 0.148) and f ^*^ = (0.139, 0.154) at T^*^ = 0.5 and T^*^ = −0.5, respectively. Same trend and almost overlapped frequency ranges of full-wave bandgaps can be found in the dispersion curves of the infinite metastructure under the same control torques, as shown in Fig. 6b and c, which successfully validate the tunability of the finite metastructures. The lonely peaks in the FRF_C_ valleys are due to the finite structure resonant motions. Second, it is noticed that no second FRF valley can be found for T^*^ = 0.5, which indicates that the on/off bandgap switch ability can also be found in the finite metastructure. Finally, three cutoff frequencies can be found in the FRFs results at the frequency points f ^*^ = 0.281, f ^*^ = 0.235 and f ^*^ = 0.315 for the T^*^ = 0, T^*^ = 0.5 and T^*^ = −0.5, respectively. Comparing with the cutoff frequencies predicted in Fig. 6b and c, very good agreements can be observed.

## Discussion

A theoretical model with coupled axial-torsional effective stiffness is developed to study the band structures of metastructures consisting of prismatic tensegrity cells. Various unit cell designs are conducted based on Bragg scattering mechanism to build tensegrity metastructures with bandgaps at desired frequency ranges. It is noticed that unit cell with opposite chirality can lead to broadband attenuations for both axial and torsional waves. Moreover, tunable wave propagations are investigated by two approaches: (i) harnessing the geometrically nonlinear deformation of the periodical tensegrity prisms under global control torques to achieve ‘small-on-large’ tunability; (ii) adjusting the prestress in the tensegrity strings with active components to achieve fine adjustment of the band structure. Finally, frequency responses studies on the finite structures are preformed to validate the wave attenuation ability as well as the tunability of the proposed tensegrity metastructures.

## Methods

### Static geometric nonlinear behavior of PTS

In Fig. 1b, the equilibrium equation at joint A′ can be expressed by using a local coordinate system *n*-*t*-*z* as:

1 $${p}_{s}\frac{({\bf{O}}{\bf{A}}^{\prime} -{\bf{O}}{\bf{B}})}{|{\bf{O}}{\bf{A}}^{\prime} {\boldsymbol{-}}{\bf{O}}{\bf{B}}{\boldsymbol{|}}}+{p}_{b}\frac{({\bf{O}}{\bf{A}}^{\prime} -{\bf{O}}{\bf{A}})}{|{\bf{O}}{\bf{A}}^{\prime} {\boldsymbol{-}}{\bf{O}}{\bf{A}}{\boldsymbol{|}}}+{p}_{e}\frac{({\bf{O}}{\bf{A}}^{\prime} -{\bf{O}}{\bf{B}}^{\prime} )}{|{\bf{O}}{\bf{A}}^{\prime} {\boldsymbol{-}}{\bf{O}}{\bf{B}}^{\prime} {\boldsymbol{|}}}+{p}_{e}\frac{({\bf{O}}{\bf{A}}^{\prime} -{\bf{O}}{\bf{C}}^{\prime} )}{|{\bf{O}}{\bf{A}}^{\prime} {\boldsymbol{-}}{\bf{O}}{\bf{C}}^{\prime} {\boldsymbol{|}}}={\bf{f}}$$

where, ${\bf{f}}=({f}_{n},{f}_{t},{f}_{z}\,)$ is the force applied at A′, *p*_s_, *p*_b_ and *p*_e_ are the magnitudes of the forces along the side string, the bar and the end string, respectively. The unit direction vectors in Eq. (1) are defined as

2 $$\begin{array}{ccc}\frac{{\bf{O}}{\bf{A}}^{\prime} {\boldsymbol{-}}{\bf{O}}{\bf{A}}}{|{\bf{O}}{\bf{A}}^{\prime} {\boldsymbol{-}}{\bf{O}}{\bf{A}}{\boldsymbol{|}}} & = & \frac{[R-R\,\cos (\varphi )]}{\sqrt{{R}^{2}[2-2\,\cos (\varphi )]+{h}^{2}}}{\bf{n}}+\frac{R\,\sin (\varphi )}{\sqrt{{R}^{2}[2-2\,\cos (\varphi )]+{h}^{2}}}{\bf{t}}\\  &  & +\,\frac{h}{\sqrt{{R}^{2}[2-2\,\cos (\varphi )]+{h}^{2}}}{\bf{z}}\\ \frac{{\bf{O}}{\bf{A}}^{\prime} {\boldsymbol{-}}{\bf{O}}{\bf{B}}}{|{\bf{O}}{\bf{A}}^{\prime} {\boldsymbol{-}}{\bf{O}}{\bf{B}}|} & = & \frac{[R-R\,\cos (\frac{2}{3}\pi -\varphi )]}{\sqrt{{R}^{2}[2-2\,\cos (\frac{2}{3}\pi -\varphi )]+{h}^{2}}}{\bf{n}}-\frac{R\,\sin (\frac{2}{3}\pi -\varphi )}{\sqrt{{R}^{2}[2-2\,\cos (\frac{2}{3}\pi -\varphi )]+{h}^{2}}}{\bf{t}}\\  &  & +\,\frac{h}{\sqrt{{R}^{2}[2-2\,\cos (\frac{2}{3}\pi -\varphi )]+{h}^{2}}}{\bf{z}}\\ \frac{({\bf{O}}{\bf{A}}^{\prime} {\boldsymbol{-}}{\bf{O}}{\bf{B}}^{\prime} )}{|{\bf{O}}{\bf{A}}^{\prime} {\boldsymbol{-}}{\bf{O}}{\bf{B}}^{\prime} |}+\frac{({\bf{O}}{\bf{A}}^{\prime} {\boldsymbol{-}}{\bf{O}}{\bf{C}}^{\prime} )}{|{\bf{O}}{\bf{A}}^{\prime} {\boldsymbol{-}}{\bf{O}}{\bf{C}}^{\prime} |} & = & {\bf{n}}\end{array}$$

Eq. (1) can also be written in a matrix form as

3 $${\bf{CP}}={\bf{f}}$$

where

$$\begin{array}{rcl}{\bf{C}} & = & [\begin{array}{ccc}\frac{R-R\,\cos (\varphi )}{\sqrt{{R}^{2}[2-2\,\cos (\varphi )]+{h}^{2}}} & \frac{R\,\sin (\varphi )}{\sqrt{{R}^{2}[2-2\,\cos (\varphi )]+{h}^{2}}} & \frac{h}{\sqrt{{R}^{2}[2-2\,\cos (\varphi )]+{h}^{2}}}\\ \frac{R-R\,\cos (\frac{2}{3}\pi -\varphi )}{\sqrt{{R}^{2}[2-2\,\cos (\frac{2}{3}\pi -\varphi )]+{h}^{2}}} & \frac{-R\,\sin (\frac{2}{3}\pi -\varphi )}{\sqrt{{R}^{2}[2-2\,\cos (\frac{2}{3}\pi -\varphi )]+{h}^{2}}} & \frac{h}{\sqrt{{R}^{2}[2-2\,\cos (\frac{2}{3}\pi -\varphi )]+{h}^{2}}}\\ 1 & 0 & 0\end{array}],\\ {\bf{P}} & = & \{\begin{array}{c}{{p}}_{{\rm{s}}}\\ {{p}}_{{\rm{b}}}\\ {{p}}_{{\rm{t}}}\end{array}\},\,{\bf{f}}=\{\begin{array}{c}{{f}}_{{\rm{n}}}\\ {{f}}_{{\rm{t}}}\\ {{f}}_{{\rm{z}}}\end{array}\}.\end{array}$$

when the PTS is unloaded, the point force **f** should be **0**. Such condition requires that the homogeneous equation **CP** = **0** should have a nontrivial solution so that *p*_s_ is positive and no string is under compression. As a result, the determinant of the matrix **C** should be zero as

4 $${\rm{\det }}({\bf{C}})=0$$

which results in two possible equilibrium configurations with *ϕ*_0_ = −π/6 or 5π/6. However, *ϕ*_0_ = −π/6 is an unstable equilibrium configuration since the system at this position always yields a maximum of potential energy^41^. Therefore, the only stable equilibrium configuration permits *ϕ*_0_ = 5π/6. Then, the global coordinates of the three joints in Fig. 1b can be expressed as:

5 $$\begin{array}{c}{\rm{Joint}}\,{\rm{A}}:\,{{\rm{x}}}_{{\rm{A}}}={R},\,{{\rm{y}}}_{{\rm{A}}}=0,\,{{\rm{z}}}_{{\rm{A}}}=0\\ {\rm{Joint}}\,{\rm{B}}:\,{{\rm{x}}}_{{\rm{B}}}={R}\,\cos (2/3\pi ),\,{{\rm{y}}}_{{\rm{B}}}={R}\,\sin (2/3\pi ),\,{{\rm{z}}}_{{\rm{B}}}=0\\ {\rm{Joint}}\,{\rm{A}}^{\prime} :\,{{\rm{x}}}_{{\rm{A}}^{\prime} }={R}\,\cos ({\varphi }_{{\boldsymbol{0}}}+\theta ),\,{{\rm{y}}}_{{\rm{A}}^{\prime} }={R}\,\sin ({\varphi }_{{\boldsymbol{0}}}+\theta ),\,{{\boldsymbol{z}}}_{{\rm{A}}^{\prime} }={{h}}_{0}+{u}\end{array}$$

where ${h}_{0}=h(u=0,\theta =0)$. The current length of the bars and strings can be given based on the coordinates of the joints as

6 $$\begin{array}{c}{{L}}_{{b}}=\sqrt{{[{R}\cos ({\varphi }_{0}+\theta )-{R}]}^{2}+{[{R}\sin ({\varphi }_{0}+\theta )]}^{2}+{({{h}}_{0}+{u})}^{2}}\\ {{L}}_{{s}}=\sqrt{{[{R}\cos ({\varphi }_{0}+\theta )-R\cos (2/3\pi )]}^{2}+{[{R}\sin ({\varphi }_{0}+\theta )-{R}\sin (2/3{\rm{\pi }})]}^{2}+{({{h}}_{0}+{u})}^{2}}\end{array}$$

Therefore, the prestresses in the bars and strings can be calculated as:

7 $$\begin{array}{c}{p}_{{\rm{b}}}={k}_{{\rm{b}}}({L}_{{\rm{b}}}^{{\rm{0}}}-{L}_{{\rm{b}}}^{{\rm{n}}})\\ {p}_{{\rm{s}}}={k}_{{\boldsymbol{s}}}({L}_{{\rm{s}}}^{{\rm{0}}}-{L}_{{\rm{s}}}^{{\rm{n}}})\end{array}$$

where ${L}_{{\rm{b}}}^{{\rm{0}}}={L}_{{\rm{b}}}(u=0,\theta =0)$ and ${L}_{{\rm{s}}}^{{\rm{0}}}={L}_{{\rm{s}}}(u=0,\theta ={\rm{0}})$; *k*_*b*_ is the stiffness of the bar and *k*_s_ is the stiffness of the string; ${L}_{{\rm{b}}}^{{\rm{n}}}$ and ${L}_{{\rm{s}}}^{{\rm{n}}}$ are the nature length(rest length) of the bar and the string, respectively.

For the tensegrity structure under external axial force and torque, the potential energy of the tensegrity structure can be calculated as:

8 $$E=\frac{3}{2}{k}_{{\rm{b}}}{\rm{\Delta }}{{L}_{{\rm{b}}}}^{2}+\frac{3}{2}{k}_{{\rm{s}}}{\rm{\Delta }}{{L}_{{\rm{s}}}}^{2}$$

where Δ*L*_b_ = *L*_b_ − ${L}_{{\rm{b}}}^{{\rm{n}}}$ and Δ*L*_s_ = *L*_s_ − ${L}_{{\rm{b}}}^{{\rm{n}}}$ are the length changes of the bars and strings, respectively. The externally applied torque *T* and the externally applied force *F* are given in Eq. (9) and Eq. (10), respectively. The *F* and *T* as functions of *u* and *θ* are shown in Fig. 1c and d, respectively.

9 $$\begin{array}{rcl}F(u,\theta ) & = & \frac{\partial E}{\partial u}=3({h}_{0}+u)[{k}_{{\rm{b}}}+{k}_{{\rm{s}}}-\frac{{k}_{b}{L}_{{\rm{b}}}^{{\rm{n}}}}{\sqrt{{h}_{0}^{2}+2{R}^{2}+2{h}_{0}u+{u}^{2}-2{R}^{2}\,\cos (\theta )}}\\  &  & -\frac{{k}_{{\rm{s}}}{L}_{{\rm{s}}}^{{\rm{n}}}}{\sqrt{{h}_{0}^{2}+2{R}^{2}+2{h}_{{\rm{0}}}u+{u}^{2}-{R}^{2}\,\cos (\theta )-\sqrt{3}{R}^{2}\,\sin (\theta )}}]\end{array}$$

10 $$\begin{array}{rcl}T(u,\theta ) & = & \frac{\partial E}{\partial \theta }=3{R}^{2}[{k}_{{\rm{b}}}\,\sin (\theta )-{k}_{{\rm{s}}}\,\cos (\frac{\pi }{6}-\theta )-\frac{{L}_{{\rm{b}}}^{{\rm{n}}}\,\sin (\theta )}{\sqrt{{h}_{0}^{2}+2{R}^{2}+2{h}_{0}u+{u}^{2}-2{R}^{2}\,\cos (\theta )}}\\  &  & \,+\frac{{k}_{s}{L}_{{\rm{s}}}^{{\rm{n}}}\,\cos (\frac{\pi }{6}-\theta )}{\sqrt{{h}_{0}^{2}+2{R}^{2}+2{h}_{0}u+{u}^{2}+{R}^{2}\,\cos (\theta )-\sqrt{3}{R}^{2}\,\sin (\theta )}}]\end{array}$$

By taking the partial derivatives of *T* and *F*, the effective tangent stiffness can be obtained as nonlinear functions of *u*_0_ and *θ*_0_. Where *k*_h_ and *k*_c_ are the slopes of the *u*-directional tangent and *θ*-directional tangent in Fig. 1c, respectively. Similarly, *k*′_c_ and *k*_m_ are the slopes of the *u*-directional tangent and *θ*-directional tangent in Fig. 1d, respectively. The schematics of the physical significance of *k*_h_, *k*_c_, *k*′_c_ and *k*_m_ are also provided in Fig. 1c and d. From the calculation results, it is found that *k*′_c_*= k*_c_ and therefore, *k*_h_, *k*_c_ and *k*_m_ are named as the effective tangent axial stiffness, effective tangent coupling stiffness and effective tangent rotation stiffness, respectively.

11 $$\begin{array}{rcl}{k}_{{\rm{h}}}({u}_{0},{\theta }_{0}) & = & \frac{\partial F}{\partial u}({u}_{0},{\theta }_{{\boldsymbol{0}}})=\frac{3{k}_{{\rm{b}}}\{\,-\,2{L}_{{\rm{b}}}^{{\rm{n}}}{R}^{2}+2{L}_{{\rm{b}}}^{{\rm{n}}}{R}^{2}\,\cos ({\theta }_{{\boldsymbol{0}}})+{[{h}_{0}^{2}+2{R}^{2}+2{h}_{{\rm{0}}}{u}_{0}+{u}_{0}^{2}-2{R}^{2}\cos ({\theta }_{{\boldsymbol{0}}})]}^{3/2}\}}{{[{h}_{0}^{2}+2{R}^{2}+2{h}_{0}{u}_{0}+{u}_{0}^{2}-2{R}^{2}\cos ({\theta }_{{\boldsymbol{0}}})]}^{3/2}}\\  &  & +\frac{3{k}_{{\rm{s}}}[\,-\,2{L}_{{\rm{s}}}^{{\rm{n}}}{R}^{2}-{L}_{{\rm{s}}}^{{\rm{n}}}{R}^{2}\,\cos ({\theta }_{{\boldsymbol{0}}})+\sqrt{3}{L}_{{\rm{s}}}^{{\rm{n}}}{R}^{2}\,\sin ({\theta }_{{\boldsymbol{0}}})+{({h}_{0}^{2}+2{R}^{2}+2{h}_{0}{u}_{0}+{u}_{0}^{2}+{R}^{2}\cos ({\theta }_{{\boldsymbol{0}}})-\sqrt{3}{R}^{2}\sin ({\theta }_{{\boldsymbol{0}}}))}^{3/2}]}{{[{h}_{0}^{2}+2{R}^{2}+2{h}_{0}{u}_{0}+{u}_{0}^{2}+{R}^{2}\cos ({\theta }_{{\boldsymbol{0}}})-\sqrt{3}{R}^{2}\sin ({\theta }_{{\boldsymbol{0}}})]}^{3/2}}\end{array}$$

12 $$\begin{array}{rcl}{k}_{{\rm{c}}}({u}_{0},{\theta }_{{\boldsymbol{0}}}) & = & \frac{\partial F}{\partial \theta }({u}_{0},{\theta }_{{\boldsymbol{0}}})={k}_{{\boldsymbol{c}}^{\prime} }({u}_{0},{\theta }_{{\boldsymbol{0}}})=\frac{\partial T}{\partial u}({u}_{0},{\theta }_{{\boldsymbol{0}}})=\frac{-3{k}_{{\rm{s}}}({h}_{0}+{u}_{0})({R}^{2}\,\cos (\pi /6-{\theta }_{{\boldsymbol{0}}}))}{[{({h}_{0}+{u}_{0})}^{{\rm{2}}}+{R}^{2}(2+\,\cos ({\theta }_{{\boldsymbol{0}}})-\sqrt{3}\,\sin ({\theta }_{{\boldsymbol{0}}}))]}\\  &  & +\frac{3{k}_{{\rm{s}}}{R}^{2}({h}_{0}+{u}_{0})\cos (\pi /6-\theta )(\,-\,{L}_{{\rm{s}}}^{{\rm{n}}}+\sqrt{{h}_{0}^{2}+2{R}^{2}+2{h}_{0}{u}_{0}+{u}_{0}^{2}+{R}^{2}\,\cos ({\theta }_{{\boldsymbol{0}}})-\sqrt{3}{R}^{2}\,\sin ({\theta }_{{\boldsymbol{0}}})}}{{[{h}_{0}^{2}+2{R}^{2}+2{h}_{0}{u}_{0}+{u}_{0}^{2}+{R}^{2}\cos ({\theta }_{{\boldsymbol{0}}})-\sqrt{3}{R}^{2}\sin ({\theta }_{{\boldsymbol{0}}})]}^{3/2}}\\  &  & +\frac{3{k}_{{\rm{b}}}{L}_{{\rm{b}}}^{{\rm{n}}}{R}^{2}({h}_{0}+{u}_{0})\sin ({\theta }_{{\boldsymbol{0}}})}{{[{h}_{0}^{2}+2{R}^{2}+2{h}_{0}{u}_{0}+{u}_{0}^{2}-{\rm{2}}{R}^{2}\cos ({\theta }_{{\boldsymbol{0}}})]}^{3/2}}\end{array}$$

13 $$\begin{array}{rcl}{k}_{{\rm{m}}}({u}_{0},{\theta }_{{\boldsymbol{0}}}) & = & \frac{\partial T}{\partial \theta }({u}_{0},{\theta }_{{\boldsymbol{0}}})=3{R}^{2}\,\cos ({\theta }_{{\boldsymbol{0}}})({k}_{{\rm{b}}}-\frac{{k}_{{\rm{b}}}{L}_{{\rm{b}}}^{{\rm{n}}}}{\sqrt{{h}_{{\boldsymbol{0}}}+2{R}^{2}+2{h}_{{\boldsymbol{0}}}{u}_{0}+{u}_{0}^{2}-2{R}^{2}\,\cos ({\theta }_{{\boldsymbol{0}}})}})\\  &  & +\,\frac{3{R}^{4}{k}_{{\rm{b}}}{L}_{{\rm{b}}}^{{\rm{n}}}{\sin }^{2}({\theta }_{{\boldsymbol{0}}})}{{[{h}_{0}^{2}+2{R}^{2}+2{h}_{0}{u}_{0}+{u}_{0}^{2}-2{R}^{2}\cos ({\theta }_{{\boldsymbol{0}}})]}^{3/2}}-3{R}^{2}{k}_{{\rm{s}}}\,\sin (\pi /6-{\theta }_{{\boldsymbol{0}}})\\  &  & +\,\frac{3{R}^{4}{k}_{{\rm{s}}}{L}_{{\rm{s}}}^{{\rm{n}}}{\cos }^{2}(\pi /6-{\theta }_{{\boldsymbol{0}}})}{{[{h}_{0}^{2}+2{R}^{2}+2{h}_{0}{u}_{0}+{u}_{0}^{2}+{R}^{2}\cos ({\theta }_{{\boldsymbol{0}}})-\sqrt{3}{R}^{2}\sin ({\theta }_{{\boldsymbol{0}}})]}^{3/2}}\\  &  & +\,\frac{3{R}^{2}{k}_{{\boldsymbol{s}}}{L}_{{\boldsymbol{s}}}^{{\boldsymbol{n}}}sin(\pi /6-{\theta }_{{\boldsymbol{0}}})}{\sqrt{{h}_{0}^{2}+2{R}^{2}+2{h}_{0}{u}_{0}+{u}_{0}^{2}+{R}^{2}\,\cos ({\theta }_{{\boldsymbol{0}}})-\sqrt{3}{R}^{2}\,\sin ({\theta }_{{\boldsymbol{0}}})}}\end{array}$$

### Dynamic linearization of 1D infinite PTS chain

For elastic wave propagations in 1D infinite PTS chain shown in Fig. 2a, an equilibrium analysis in the *n*^th^ hollow disk is conducted, as shown in Fig. 2b, and the governing equation of the *n*^th^ hollow disk can be expressed as

14 $$\begin{array}{rcc}m{\ddot{u}}_{{\rm{n}}} & = & F({u}_{{\rm{n}}+1}-{u}_{{\rm{n}}},{\theta }_{{\rm{n}}+1}-{\theta }_{{\rm{n}}})-F({u}_{{\rm{n}}}-{u}_{{\rm{n}}-1},{\theta }_{{\rm{n}}}-{\theta }_{{\rm{n}}-1})\\ {J}{\ddot{\theta }}_{{\rm{n}}} & = & T({u}_{{\rm{n}}+1}-{u}_{{\rm{n}}},{\theta }_{{\rm{n}}+1}-{\theta }_{{\rm{n}}})-T({u}_{{\rm{n}}}-{u}_{{\rm{n}}-1},{\theta }_{{\rm{n}}}-{\theta }_{{\rm{n}}-1})\end{array}$$

where *m* and *J* are the mass and moment of inertia of the Ti hollow disk, respectively. Both *F* and *T* are nonlinear functions which are mentioned in Eq. (9) and Eq. (10).

First, we consider an incremental dynamic deformation superimposed upon an equilibrium state, which is defined as *u*_0_ and *θ*_0_. The Incremental dynamic deformations are given by Δ*u*(t), Δ*θ*(t), with Δ denoting a small increment in the quantity concerned. Eq. (14) can then be linearized with first order Taylor expansion as

15 $$\begin{array}{rcc}m{\rm{\Delta }}{\ddot{u}}_{{\rm{n}}} & = & {k}_{{\rm{h}}}({u}_{{\boldsymbol{0}}},{\theta }_{{\boldsymbol{0}}})({\rm{\Delta }}{u}_{{\rm{n}}+1}+{\rm{\Delta }}{u}_{{\rm{n}}-1}-2{\rm{\Delta }}{u}_{{\rm{n}}})+{k}_{{\rm{c}}}({u}_{{\boldsymbol{0}}},{\theta }_{{\boldsymbol{0}}})({\rm{\Delta }}{\theta }_{{\rm{n}}+1}+{\rm{\Delta }}{\theta }_{{\rm{n}}-1}-2{\rm{\Delta }}{\theta }_{{\rm{n}}})\\ {J}{\rm{\Delta }}{\ddot{\theta }}_{{\rm{n}}} & = & {k}_{{\rm{c}}}({u}_{{\boldsymbol{0}}},{\theta }_{{\boldsymbol{0}}})({\rm{\Delta }}{u}_{{\rm{n}}+1}+{\rm{\Delta }}{u}_{{\rm{n}}-1}-2{\rm{\Delta }}{u}_{{\rm{n}}})+{k}_{{\rm{m}}}({u}_{{\boldsymbol{0}}},{\theta }_{{\boldsymbol{0}}})({\rm{\Delta }}{\theta }_{{\rm{n}}+1}+{\rm{\Delta }}{\theta }_{{\rm{n}}-{\boldsymbol{1}}}-2{\rm{\Delta }}{\theta }_{{\rm{n}}})\end{array}$$

Considering harmonic small-amplitude wave excitations with angular frequency *ω* and using the Bloch theorem, Δ*u*(t), Δ*θ*(t) can be written as

16 $$\begin{array}{rcc}{\rm{\Delta }}{u}_{n} & = & {\rm{\Delta }}{u}_{1}{e}^{inkh}={C}_{1}{e}^{i(nkh-\omega t)}\\ {\rm{\Delta }}{\theta }_{n} & = & {\rm{\Delta }}{\theta }_{1}{e}^{inkh}={C}_{2}{e}^{i(nkh-\omega t)}\end{array}$$

Then, the governing equation can be rewritten as

17 $${\omega }^{2}[\begin{array}{cc}m & 0\\ 0 & J\end{array}]\{\begin{array}{c}{{C}}_{1}\\ {{C}}_{2}\end{array}\}=[\begin{array}{cc}{k}_{{\rm{h}}}({e}^{ikh}+{e}^{-ikh}-2) & {k}_{{\rm{c}}}({e}^{ikh}+{e}^{-ikh}-2)\\ {k}_{{\rm{c}}}({e}^{ikh}+{e}^{-ikh}-2) & {k}_{{\rm{m}}}({e}^{ikh}+{e}^{-ikh}-2)\end{array}]\{\begin{array}{c}{{C}}_{1}\\ {{C}}_{2}\end{array}\}$$

where *C*_1_ and *C*_2_ are the amplitudes of the axial and rotational displacements in the first PTS unit cell, respectively. *k* is the wavenumbers. An eigenvalue problem is then formed from Eq. (17) and the dispersion results for two wave modes can be calculated.
